# CTSB Knockdown Inhibits Proliferation and Tumorigenesis in HL-60 Cells

**DOI:** 10.7150/ijms.54206

**Published:** 2021-01-29

**Authors:** Sida Peng, Qingqing Yang, Huan Li, Yuhang Pan, Jiani Wang, Pan Hu, Nana Zhang

**Affiliations:** 1 Department of Hematology, The First Affiliated Hospital of Guangzhou Medical University, Guangzhou, 510230, China.; 2 Department of Clinical Laboratory, The First Affiliated Hospital of Guangzhou Medical University, Guangzhou, 510230, China.; 3 Breast Cancer Center, The Third Affiliated Hospital of Sun Yat-sen University, Guangzhou, 510000, China.; 4 Cell genetics laboratory, The First Affiliated Hospital of Guangzhou Medical University, Guangzhou, 510230, China.; 5 Department of Pathology, the Third Affiliated Hospital of Sun Yat-sen University, Guangzhou510000, P. R. China.

**Keywords:** CTSB, proliferation, tumorigenesis, AML, AKT pathway.

## Abstract

**Background:** Cathepsin B (CTSB) was well documented in solid tumors, up-regulated of CTSB expression is linked with progression of tumors. However, the study of CTSB in adult leukemia has not been reported. **Methods:** Total RNA was isolated from PBMC (peripheral blood mononuclear cell) of AML patients and healthy donors. qRT-PCR was performed to detect the expression of CTSB. The association of CTSB expression with the patients' overall survival (OS) and disease-free survival (DFS) were analyzed. Stable HL-60 CTSB-shRNA cell lines were established by retrovirus infection and puromycin selection. Cell proliferation was detected by CCK-8 analysis. Tumorigenesis ability was analyzed by soft agar and xenograft nude mice model. Western blot was performed to detect the expression of CTSB and the proteins of cell signaling pathway. **Results:** The mRNA expression level of CTSB was up-regulated in AML patients compared to healthy control (*p*<0.001), and CTSB expression was significantly higher in M1, M2, M4 and M5 AML samples than healthy control. The CTSB expression in AML was associated with WBC count (*p*=0.037). Patients with high CTSB expression had a relatively poor OS (*p*=0.007) and a shorter DFS (*p*=0.018). Moreover, the expression level of CTSB may act as an independent prognostic factor for both OS (*p*=0.011) and DFS (*p*=0.004). Knockdown CTSB expression in HL-60 cells could inhibit the cells' proliferation and tumorigeneses *in vitro* and *in vivo*. Further study showed knockdown CTSB expression in HL-60 cells could inactive the AKT signaling pathway. **Conclusions:** CTSB mRNA was upregulated in AML patients. CTSB overexpression was correlated with poor prognosis and may serve as an independent prognostic factor for both OS and DFS in AML patients. Knockdown CTSB expression in HL-60 cells could inhibit the cells' proliferation and tumorigenesis. The underlying mechanism may be the inhibition of the AKT signaling pathway.

## Introduction

Acute myeloid leukemia (AML) is now recognized as a hematological malignancy characterized by the blockage of myeloid differentiation and uncontrolled clonal proliferation 1. AML, poses a significant clinical burden in the worldwide, and is the most common type of leukemia in adults with an incidence of 3.7 per 100,000 people. Based on the newly update data of leukemia, the incidence of AML in USA is 5.2 per 100,000 for males, 3.6 per 100,000 for females 2. Despite recent advance in the AML, the overall survival rate remains <50% 3, 4. Further studies are required eagerly to clarify the underlying mechanisms of AML and provide novel biomarker for prognosis and treatment. In view of the fact that accurate genetic examinations in treatment and prognosis of AML patients 5, it is important to develop novel biomarkers related to AML.

Cathepsin B (CTSB) was primarily found as a cysteine protease that was involved in the degradation of lysosomal proteins 6. Over expression of CTSB has been reported in numerous types of solid tumors, including salivary adenoid cystic carcinoma, brain tumor, breast tumor, colorectal tumor, and so on 7-10. We have reported CTSB was overexpressed in gastric carcinoma 11 but down regulated in hepatocellular carcinoma 12. It was considered as a potential biomarker of poor prognosis 7-10.

It was reported that overexpression of CTSB could promote cell invasion and metastasis of colorectal cancer, liver cancer, gastric cancer, glioma and ovarian cancer 13-17. In addition, silence or inhibition of CTSB expression has been reported to inhibit cell proliferation, including endometrial cancer cells, meningioma cells, and human T cells 18-20. Here we reported that CTSB mRNA was upregulated in AML peripheral blood samples and related to patient's poor prognosis. Then CTSB functions were investigated using the HL-60 AML cell line by knockdown the expression of CTSB. Our results showed that knockdown CTSB by shRNA could decrease cell proliferation and tumorigenesis in HL-60 cells. The analysis of pathway signals showed that knockdown CTSB in HL-60 cells could decrease the expression of p-AKT. So decreased CTSB expression repressed the cell proliferation and tumorigenesis maybe through AKT inactivation in HL-60 cells.

## Materials and Methods

### Patients and specimens

Eighty seven newly diagnosed AML patients from Aug 2010 to Jun 2014 and 36 healthy control at the First Affiliated Hospital of Guangzhou Medical University were enrolled in this study. AML and healthy control peripheral blood samples were collected and mononuclear cells were isolated by Ficoll-Hypaque gradient separation according the manufacturer protocol. Characteristics of AML patients are described in Table 1. The follow-up time of the AML patients ranged from 1 to 119 months. The median follow-up time was up to 35 months. With the informed consent of the patients, the peripheral blood samples of AML patients were collected before anti-leukemia treatment. The study was approved by the Ethics Committee of the first affiliated Hospital of Guangzhou Medical University.

### Cell culture

HL-60 cells (Cell bank of the Chinese Academy of Sciences, Shanghai) were cultured in RPMI-1640 medium (Hyclone, Logan, USA) supplemented with 10% fetal bovine serum (FBS; ThermoFisher Gibco, USA), penicillin (100 units/ml), and streptomycin (100 units/ml) maintained at 37 °C and 5% CO2 incubator.

### Reverse transcription and quantitative real-time PCR assays (qRT-PCR)

Total RNA was isolated from PBMC (peripheral blood mononuclear cell) of AML patients using TRIzol reagent (Transgen Biotech, Beijing, China), according to the protocol of manufacturer. First Strand cDNA Synthesis Kit (Thermo Fisher scientific, Waltham, MA, USA) was used to reverse transcribe RNA to cDNA according to the manufacturer's protocol. A total of 1.0 ug of mRNA was used to reverse transcribe RNA to cDNA. RT‑qPCR was carried out with SYBR Green Master Mix (Transgen Biotech, Beijing, China). The qPCR was performed at a total 10 μl and the following thermocycling conditions were used for the qPCR: Initial denaturation at 95˚C for 10 min; 40 cycles of 95˚C 15 sec and 55˚C for 20 sec, followed by a dissociation step at 95 °C for 15 s, and 60 °C for 30 s, Primer sequences were used as follows: CTSB sense 5'-GCAGGCCGGGCACAAC-3', antisense 5'-GGAGGCCCAGAGCTGCCAC-AT-3'. GAPDH sense 5'-TGTTGCCATCAATGACC-CC-3', antisense 5'-CTCCACGACGTACTCAGC-3'. The results were normalized to the expression of GAPDH.

All experiments were performed in triplicate. The relative expression of target gene was calculated by 2-ΔCt method (ΔCt = CT target gene-CTGAPDH).

### Western blot analysis

Western blot was performed as previously described 21. The blots were probed with rabbit anti-CTSB antibody (Proteintech Group, Inc., Rosemont, IL, USA), anti-AKT antibody, anti-p-AKT antibody, anti-p-ERK antibody, anti-p38 antibody, anti-p-p38 antibody (Cell signaling, Billerica, MA). Mouse anti-GAPDH antibody (Cell signaling, Billerica, MA) was used as loading control.

### Anchorage-independent cell growth

The ability of CTSB-knockdown HL-60 cell lines growing in anchorage-independence was assessed by colony formation in soft agar as previously described 22. 5000 cells were suspended in 1ml of RPMI 1640 with 0.33% agarose (Sigma), 10% FBS and plated into a well of 12-well plate. After 14 days culture, colonies were identified with a diameter > 125 μm.

### Short hairpin RNA (shRNA) constructs and retroviral infection

Stable knockdown of endogenous CTSB was performed using retrovirus constructs targeting CTSB. The shRNA target sequences were: shRNA1, 5-CCAACACGTCAC CGGAGAGAT-3; shRNA2, 5-GCTGGTCAACTATGTCAACAA-3. The synthetic oligos were cloned into the pSuper-retro-puro vector. The successful plasmid construction was verified by DNA sequencing. Production of retrovirus was performed according to the instructions in 293FT cells. HL-60 cells were subjected with infection of retrovirus expressing CTSB-shRNA1 and CTSB-shRNA2. An empty pSuper-retro-puro was used as a control (Scramble).

### Cell proliferation assay

HL-60 CTSB-shRNA1 cells, HL-60 CTSB-shRNA2 cells and HL-60 Scramble cells were seeded in a 96-well plate at a density of 2000 cells/well and maintained at 37 °C in 5% CO2 incubator for 24 h, 48h, 72h, 96h and 120h. 10 µl of the Cell Counting Kit-8 (CCK8, Transgen Biotech, Beijing, China) was added per well and the plate was incubated for 2 h at 37 °C in 5% CO2 incubator, the absorbance at 450 nm was measured on a microplate reader.

### Mice

The tumorigenic capacity was analyzed by subcutaneous injecting HL-60 Scramble cells and HL-60 CTSB-shRNA2 cells into nude mice. All the mice were bred and maintained under SPF conditions in the Department of Animal Center, the first Affiliated Hospital of Guangzhou Medical University. Animal protocol was approved by the Institutional Animal Care and Use Committee of Guangzhou Medical University. 12 healthy nude mice, which were four to six weeks old, were randomly assigned to each group. Each mouse was subcutaneous injected with 5 × 10^6^ cells in 1:1 serum free medium and Matrigel solution. Tumor growth was measured and tumor volume was calculated according to the formula: tumor volume [mm3] = (length [mm]) × (width[mm])^2^ × 0.5 23.

### Statistical analysis

Statistical analyses were performed using Statistical Program for Social Sciences 20.0 (SPSS, Chicago, IL, USA) and Graph Pad Prism 5.0 (Graph Pad Software, La Jolla, CA, USA). The correlation between the CTSB expression and the clinicopathological parameters of patients with AML was assessed by a chi-square test. The Kaplan-Meier analysis was used to evaluate the survival curve of distinct CTSB expression of AML patients. The prognostic value of CTSB on AML patients' survival was analyzed by the Cox proportional hazard model. A p-value less than 0.05 was regarded as statistically significant.

## Result

### CTSB was up-regulated in AML and correlated with clinical outcomes

**Figure 1:**
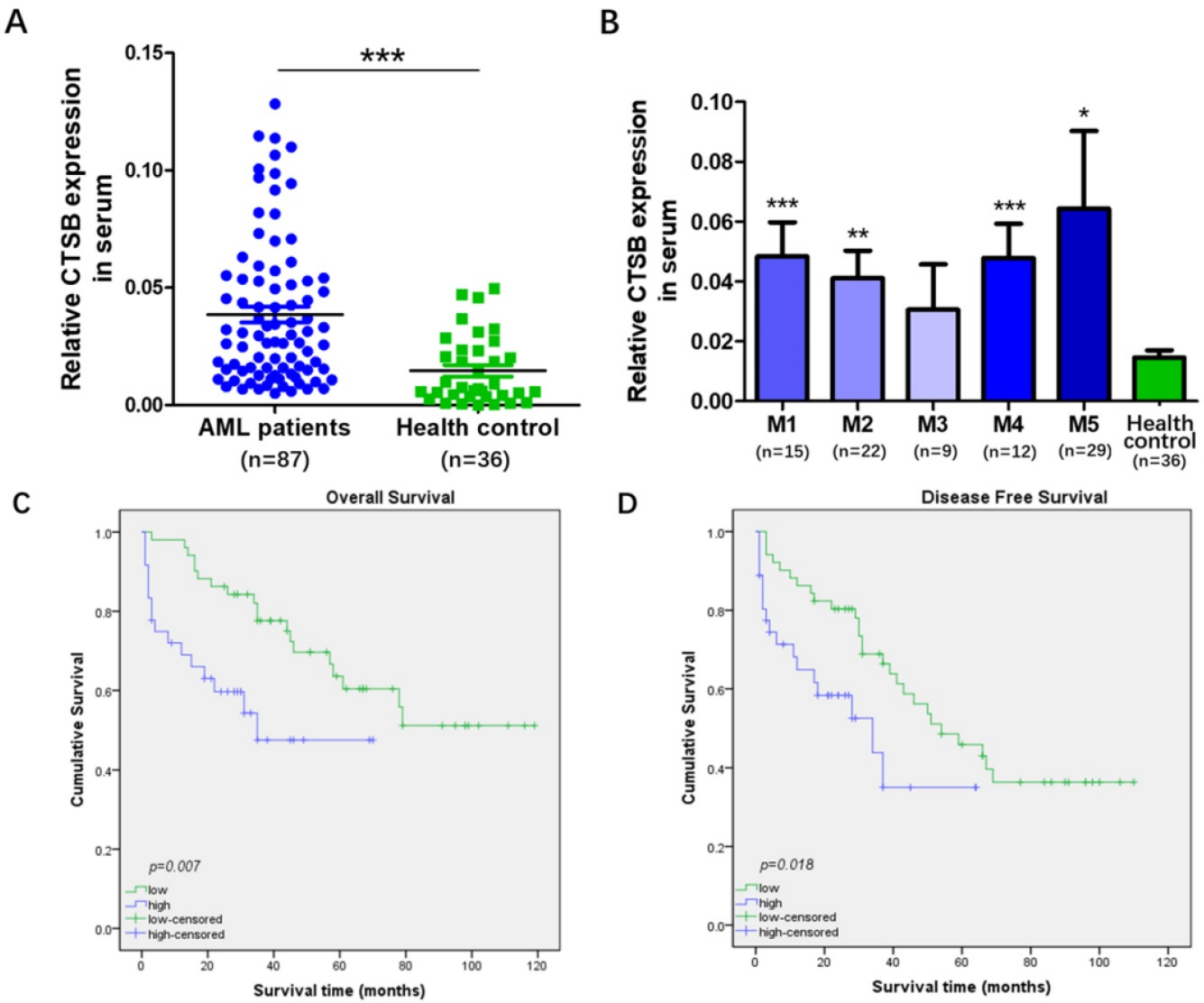
** CTSB expression was up-regulated in AML patients and related to patients' poor OS and short DFS. (A)** Expression levels of CTSB in AML patients (median value 0.039, range: 0.005-0.129) and healthy controls (median value 0.021, range: 0.001-0.055). The expression of CTSB was upregulated in AML compared with healthy controls (*p<*0.001); **(B)** Expression levels of CTSB in FAB category of AML patients showed that CTSB expression was significantly higher in M1, M2, M4 and M5 AML samples than healthy control; **(C)** The OS rate of AML patients with high and low CTSB expression (*p*=0.007); The P-value was calculated using the log-rank test. **(D)** The DFS rate of AML patients with high or low CTSB expression (*p*=0.018). The P-value was calculated using the log-rank test.

CTSB mRNA expression levels were evaluated in the peripheral blood samples of 87 newly diagnosed AML patients and 36 healthy control by qRT-PCR methods. A statistically difference was found in the mRNA expression level of CTSB between newly diagnosed AML patients and healthy control samples (p<0.001, Figure 1A). The mRNA expression of CTSB in AML patients exhibited obviously higher level (median value 0.039, range: 0.005-0.129) than healthy controls (median value 0.021, range: 0.001-0.055). We also analyzed the expression of CTSB between healthy control and FAB category of AML. CTSB expression was significantly higher in M1, M2, M4 and M5 AML samples than healthy control (Figure 1B). Moreover, correlation of CTSB expression with clinical characteristics of AML patients was analyzed. The patient samples were divided into two groups according to the expression of CTSB gene (high vs. low). High CTSB expression was detected in 36 samples (41.38%) and low CTSB expression was detected in 51 samples (58.62%, Table 1). The relationship between CTSB expression and other clinical indexes was analyzed. We found the expression of CTSB was associated with WBC count (*p*=0.037). However, there were no significant correlations between CTSB expression with patients' gender, age, FAB subtype, HGB, PLT, blast in BM and complete remission (Table 1).

### CTSB expression was correlated with OS and DFS

**Figure 2:**
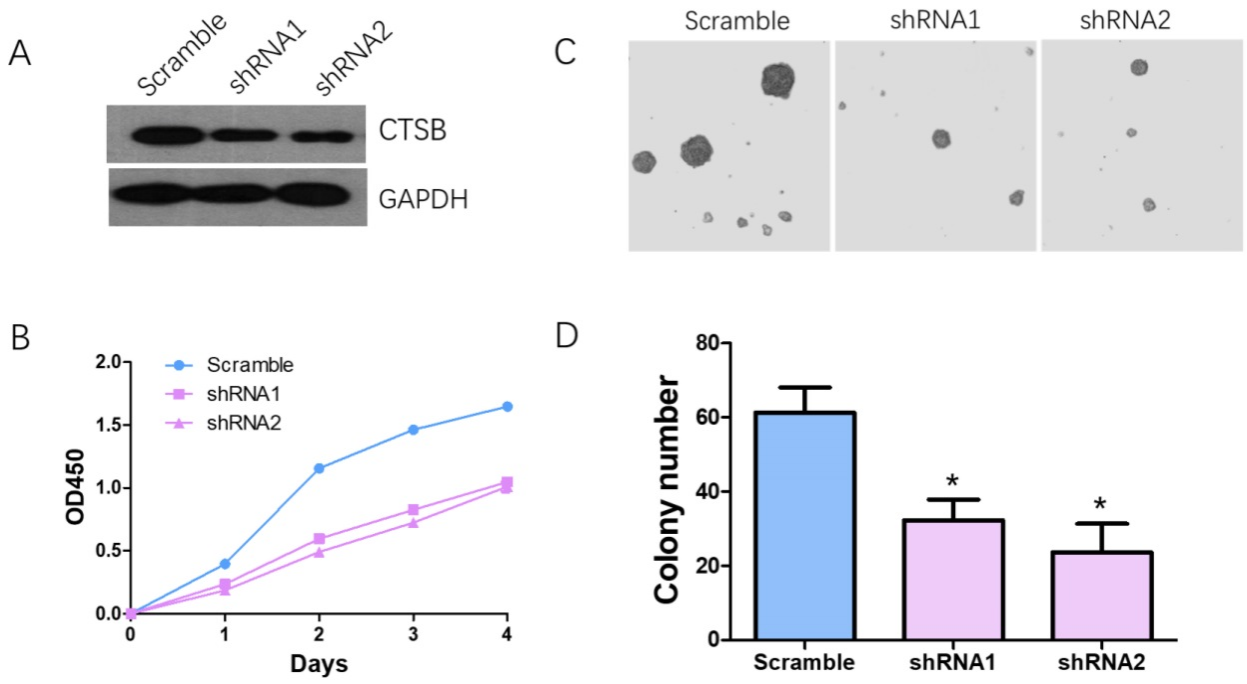
** Knockdown CTSB expression in HL-60 cells inhibit cell proliferation and colony formation *in vitro*. (A)** CTSB expression levels were detected by western blotting in HL-60 CTSB-shRNA cells and scramble cells; **(B)** CCK assay was performed. Decreased proliferation of HL-60 cells was detected after CTSB knockdown. **(C, D)** Softagar assay was performed to examine the anchorage-independent growth of HL-60 cells. Decreased colony formation ability was observed in HL-60 cells after CTSB knockdown (shRNA1 *p*=0.029, shRNA2 *p*=0.021).

Furthermore, we explored the association between the expression of CTSB and the AML patients' clinical outcomes. The data showed patients with a high expression level of CTSB had a poor overall survival (OS) (*p*=0.007) and a short disease-free survival (DFS) (*p*=0.018) (Figure 2A, B). Univariate Cox proportional hazard regression analysis showed that both blast in BM (*p*=0.001) and the CTSB expression level (*p*=0.010) were prognostic factors for OS (Table 2). The patients' age (*p* =0.003), blast in BM (*p*<0.001) and CTSB expression level (*p*=0.021) were associated with DFS (Table 3). The multivariate adjustment for the above significant clinicopathological features showed that the expression level of CTSB was an independent prognostic factor for both OS (Hazard ratio [HR] =0.393, 95% confidence interval [CI], 0.192-0.805, *p*=0.011) and DFS (Hazard ratio [HR] =0.363, 95% confidence interval [CI], 0.182-0.725, *p*=0.004) of AML patients.

### CTSB knockdown inhibits the proliferation and tumorigenesis of HL-60 cells

HL-60 cells were infected with CTSB-shRNA1, CTSB-shRNA2 retrovirus which were mediated by pSuper-retro-puro vector. Western blot analysis was used to determine the effect of CTSB knockdown. The result showed that the CTSB protein expression level was decreased in CTSB-shRNA cells (Fig. 2A). CCK-8 analysis was performed to detect the cell proliferation effect of CTSB-shRNA in HL-60 cells. As show in Figure 2B, a significant decrease in proliferation rate was observed in CTSB-shRNA1 and CTSB-shRNA2 cells compared to scramble cells, indicating that CTSB knockdown could inhibit HL-60 cells' proliferation (Fig. 2B). To further examine tumor-suppressing activity of CTSB-shRNA, softagar assay was performed to examine the anchorage-independent growth of HL-60 cells. The results showed that CTSB-shRNA could inhibit anchorage-independent growth of HL-60 cells (Fig. 2C, D).

### CTSB knockdown attenuates tumor growth of HL-60 cells in nude mice model

**Figure 3:**
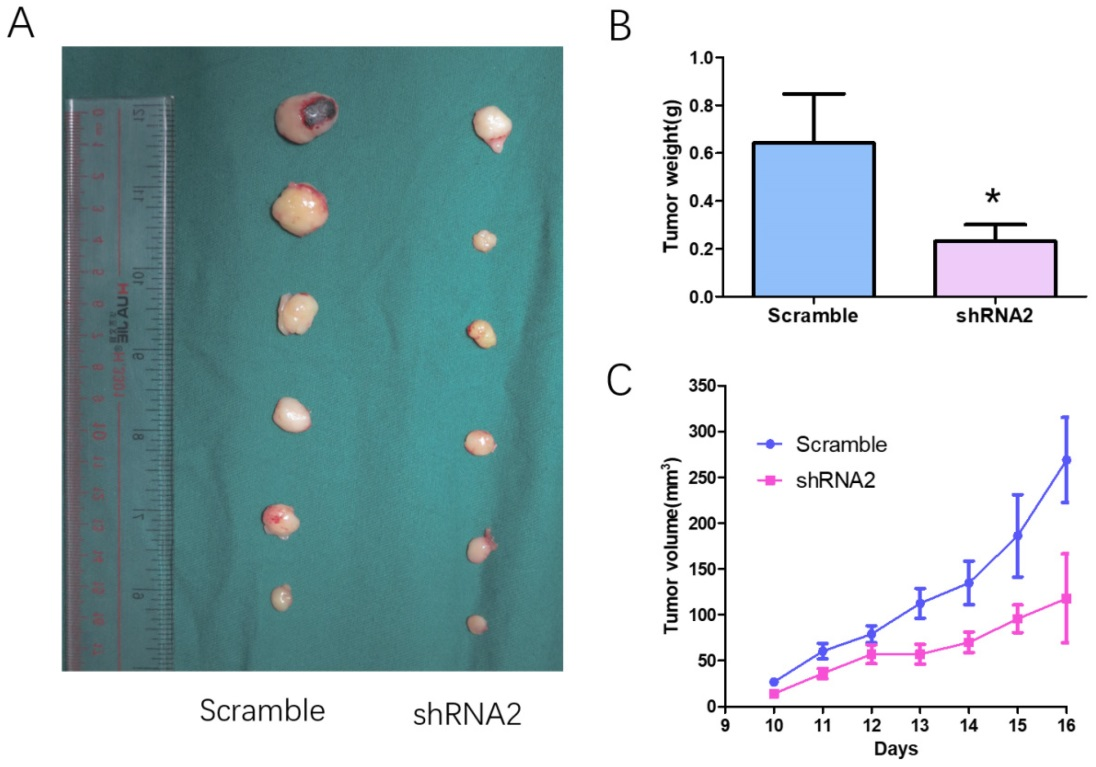
** CTSB knockdown attenuates tumor growth of HL-60 cells in nude mice model. (A)** HL-60 CTSB-shRNA2 and scramble cells were injected subcutaneously to generated tumors in mice. All mice were executive 3 weeks after injection; **(B)** Tumor size was measured. The result showed that tumor volume of CTSB-shRNA2 was decreased than scramble mice. **(C)** Tumor weight showed a decrease in CTSB-shRNA mice (*p*=0.033).

The effect of CTSB knockdown on tumor growth *in vivo* was examined in a xenograft nude mice model (Fig. 3). Mice were injected subcutaneously with 5×10^6^ individual cells infected of CTSB-shRNA1 and scramble retrovirus. Then the tumor size and weight generated by CTSB-shRNA HL-60 cells and scramble cells were compared. The results showed a smaller tumor volume and less tumor weight in CTSB-shRNA group than scramble group (Fig. 3B, C). Thus, CTSB knockdown attenuates tumor growth of HL-60 cells *in vivo*.

### CTSB may regulate HL-60 cell growth and tumorigenesis via regulation of AKT signaling pathway

**Figure 4:**
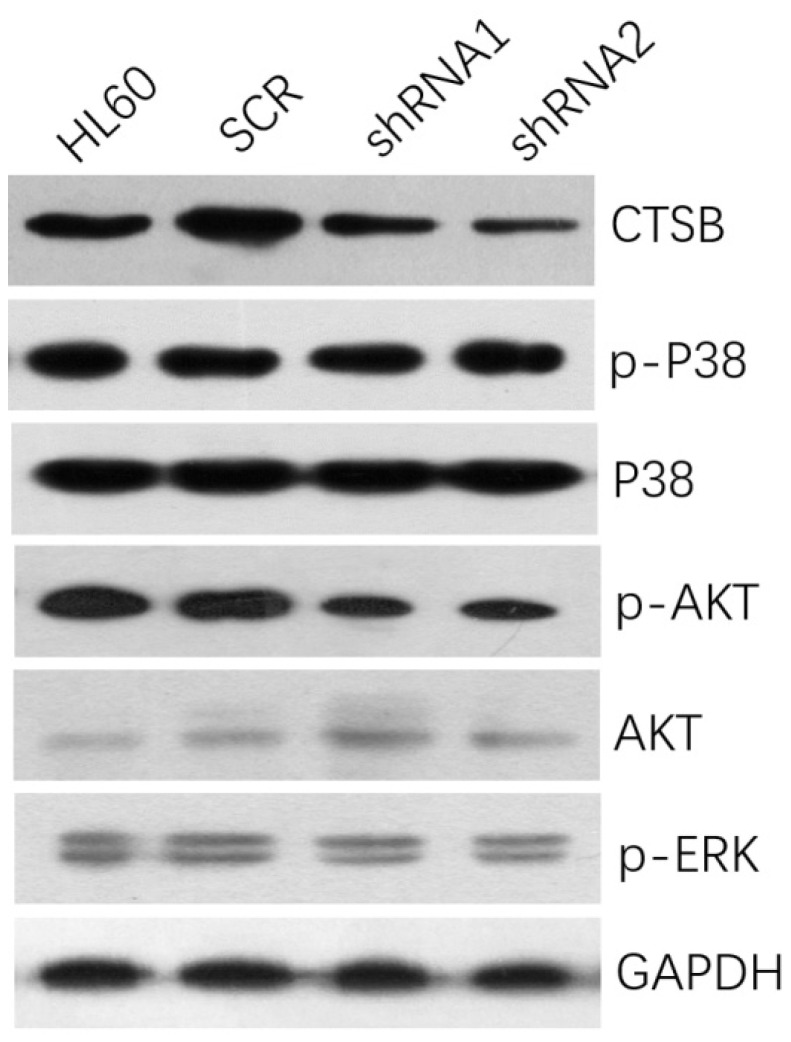
** CTSB knockdown could inhibit the expression of p-AKT.** Protein levels of p-p38, p-Akt, p-ERK were assessed in HL-60 CTSB-shRNA cells and scramble cells by western blotting, the p-AKT expression was downregulated after CTSB knockdown.

To investigate the underlying mechanisms of decreased cell proliferation and tumorigenesis of HL-60 CTSB-shRNA cells, western blot analysis was performed to detect the common tumor-related signaling pathways. We analyzed the MAPK/P38 pathway, the AKT pathway, and the ERK pathway. Then we found that when we knockdown the expression of CTSB in HL-60 cells, the p-AKT expression was downregulated, but there was no significant difference on the expressions of p-p38 and p-ERK between HL-60 shRNA cells and scramble control cells (Fig. 4). So CTSB may regulate HL-60 AML cell growth and tumorigenesis via the regulation of AKT signaling pathway.

## Discussion

Cathepsin is a lysosomal protease belonging to papain family 24. Cathepsins can be classified into serine cathepsins, cysteine cathepsins and aspartate cathepsins^25^. Human cysteine cathepsins are essential in the degradation of the proteins that are internalized in the lysosomes 26. However, cathepsins were reported not only localized endoly-sosomal compartment, they also localized in cell cytoplasm, nucleus, mitochondria and extracellular. This indicated cathepsins may have broad biological activity 27, 28. CTSB, a kind of lysosomal cysteine protease, has been reported to be a potentially effective biomarker as well as an important contributor to the progression of different types of cancers.

It was reported that CTSB might be an optimal diagnostic and prognostic marker 29-31, and CTSB can be developed to design enhanced drug delivery approaches in the recent years 32. We also reported that CTSB may serve as an optical prognostic marker in gastric carcinoma and hepatocellular carcinoma 11, 12. Many researchers presented that CTSB acted as a metastasis-related gene 7, 13, 18. The plasma membrane localization of CTSB correlated with metastatic tumors 33, 34. CTSB was also reported related to autophagy. Autophagy, a lysosomal-dependent process could prevent cancer by removing damaged proteins and reducing reactive oxygen species (ROS), and promoting the cell death. CTSB inhibition was showed to increase cell autophagy, and accumulation autophagic biomarkers could be observed 35, 36.

In this study, we found the expression of CTSB in AML patients was up-regulated compared to health control group. Patients with high expression of CTSB displayed a poor OS and DFS. Cox regression analysis showed CTSB mRNA expression was an independent prognostic factor for AML patients' OS and DFS. This result indicates the CTSB expression may act as a predictor for AML patients' prognosis and survival. Misti Jain, etc. reported that both CTSB and CTSL mRNA expression levels were up-regulated in pediatric acute myeloid leukemia 36. Total 24 pediatric AML patients were enrolled in the analysis. The result showed pediatric AML patients with higher CTSB expression exhibited an inferior DFS and OS compared to lower CTSB expression patients 36. This is the first report that CTSB was up-regulated in pediatric AML. Our report addressed CTSB expression and its clinical significance in adult AML patients. CTSB expression was up-regulated and correlated with poor prognosis of adult AML patients.

To clarify the tumor-associated functions and related mechanism of CTSB gene in AML. We generated HL-60 CTSB-shRNAs cell lines using retrovirus infection. Based on the cell model, we observed knockdown CTSB expression could result in inhibition of HL-60 cells' proliferation. Anchorage-independent cell growth analysis showed that CTSB-shRNA could inhibit anchorage-independent growth of HL-60 cells *in vitro*. Furthermore, HL-60 CTSB-shRNA cells were subcutaneously injected in mice to generated tumors. Inhibition of tumor weight and tumor volume by CTSB-shRNA was observed *in vivo*. However, we did not observe a significant change in the invasiveness of HL-60 CTSB-shRNA cells compared to control cells (data was not shown). We have not performed the cell autophagic study about CTSB in AML. It is an interesting project and we will clarify it in the next report.

At last, we assessed the AKT, p38, ERK oncogenic cell signaling pathway protein levels to found which cell signaling pathway enrolled in CTSB regulating. We found that p-AKT levels were significantly decreased after CTSB was knockdown in HL-60 cells. These findings suggested that knockdown CTSB might inhibit HL-60 cell proliferation and tumorigenesis via the inactivation of AKT signaling pathway. However, this mechanism study was simple and superficial. It only provided knockdown CTSB may inhibit HL-60 cells' proliferation and tumorigenesis through inactivation AKT signaling pathway, but we don't know how p-AKT was downregulated and AKT pathway was inactivated by CTSB-shRNA, and there is no clue if there are any other mechanism contribute to CTSB-shRNA inducing inhibition of cells' proliferation and tumorigenesis. All of these need more experiments to clarify in the future.

## Conclusions

In summary, CTSB expression was up-regulated in AML patients compare with normal control. Most importantly, patients with high CTSB expression have a poor OS and short DFS. Knockdown CTSB expression could inhibit cell proliferation and tumorigenesis in HL-60 AML cell line. The underlying mechanism may through regulating AKT oncogenic cell signaling pathway.

## Figures and Tables

**Table 1:** Correlation of CTSB expression with clinicopathologic features

Characteristics	Total (n=87)	CTSB		*P* value
		Low(n=51)	High (n=36)	
Gender				0.810
Male	47(54.0%)	27(57.4%)	20(42.6%)	
Female	40(46.0%)	24(60.0%)	16(40.0%)	
Age(years)				0.454
≤60	64(73.6%)	36(56.2%)	28(43.8%)	
>60	23(26.4%)	15(65.2%)	8(34.8%)	
FAB subtype				0.953
M1	15(17.2%)	8(53.3%)	7(46.7%)	
M 2	22(25.3%)	12(54.5%)	10(45.5%)	
M 3	9(10.3%)	6(66.7%)	3(33.3%)	
M 4	12(13.8%)	7(58.3%)	5(41.7%)	
M 5	29(33.3%)	18(62.1%)	11(37.9%)	
WBC(×10^9^/L)				0.037
<10	44(50.6%)	28(63.6%)	16(36.4%)	
≥10	43(49.4%)	23(53.5%)	20(46.5%)	
HGB(g/L)				0.988
<80	46(52.9%)	27(58.7%)	19(41.3%)	
≥80	41(47.1%)	24(58.5%)	17(41.5%)	
PLT(×10^9^/L)				0.196
<10	46(52.9%)	24(52.2%)	22(47.8%)	
≥10	41(47.1%)	27(65.9%)	14(34.1%)	
Blast in BM				0.928
<50%	43(49.4%)	25(58.1%)	18(41.9%)	
≥50%	44(50.6%)	26(59.1%)	18(40.9%)	
Complete Remission				0.498
Yes	40(46.0%)	25(62.5%)	15(37.5%)	
No	47(54.0%)	26(55.3%)	21(44.7%)	

**Table 2:** Cox-regression analysis of parameters associated with Overall survival of AML patients

Factor	Univariate		Multivariate	
	HR(95%CI)	*P* value	HR(95%CI)	*P* value
Blast in BM				
<50%	Reference		Reference	
≥50%	0.244(0.110-0.543)	0.001	0.245(0.110-0.545)	0.001
CTSB expression				
Low	Reference		Reference	
High	0.390 (0.191 -0.795)	0.010	0.393(0.192-0.805)	0.011

HR: hazard ratio. 95%CI: 95% confidence interval

**Table 3:** Cox-regression analysis of parameters associated with Disease free survival of AML patients

Factor	Univariate		Multivariate	
	HR(95%CI)	*P* value	HR(95%CI)	*P* value
Age(years)				
≤60	Reference		Reference	
>60	0.398(0.218-0.730)	0.003	0.366(0.194-0.690)	0.002
Blast in BM				
<50%	Reference		Reference	
≥50%	0.246 (0.124-0.491)	0.000	0.261(0.131 -0.521)	0.000
CTSB expression				
Low	Reference		Reference	
High	0.465(0.242 -0.893)	0.021	0.363(0.182 -0.725)	0.004

## Abbreviations

AML: acute myeloid leukemia
CTSB: Cathepsin B
PBMC: peripheral blood mononuclear cell
OS: overall survival
DFS: disease-free survival
